# Supplementary material for: Imbalance between subsets of CD8+ peripheral blood T cells in patients with chronic obstructive pulmonary disease
Source: PeerJ. 2016 Aug 2;4:e2301. doi: 10.7717/peerj.2301 (PMC4975138; doi:10.7717/peerj.2301)
Supplement: Data S1 [file peerj-04-2301-s001.pdf]

Fig. 1. circulating CD8+ T cells (mean ±SD)

	CD8+/CD3+ (%)
HN（14）	29.99±8.49
SCOPD（24）	30.36±10.25
AECOPD（14）	39.51±8.55

Fig. 2. Tc1, Tc2 and Tc17 (mean ±SD)

	Tc1 (%)	Tc2 (%)	Tc17 (%)
HN（14）	17.56±13.90	2.12±1.22	0.33±0.12
SCOPD（24）	37.10±21.26	0.76±0.69	0.38±0.38
AECOPD（14）	53.11±18.03	2.68±1.96	1.13±0.83

Fig. 3. CD8+ Tregs, Tc10 and CD8+α7+ T cells (mean ± SD)

	CD8+Tregs (%)	Tc10 (%)	CD8+α7+ (%)
HN（14）	0.76±0.48	1.06±0.34	1.03±0.91
SCOPD（24）	2.05±1.93	0.45±0.54	0.85±0.83
AECOPD（14）	2.56±2.17	0.42±0.21	0.59±0.38
